# The Peculiar Case of the Hyper‐thermostable Pyrimidine Nucleoside Phosphorylase from *Thermus thermophilus* **

**DOI:** 10.1002/cbic.202000679

**Published:** 2021-01-26

**Authors:** Felix Kaspar, Peter Neubauer, Anke Kurreck

**Affiliations:** ^1^ Department of Biotechnology, Chair of Bioprocess Engineering Technische Universität Berlin Straße des 17. Juni 135 10623 Berlin Germany; ^2^ BioNukleo GmbH Ackerstraße 76 13355 Berlin Germany

**Keywords:** cosolvent, enzymes, nucleoside phosphorylases, nucleosides, thermostable

## Abstract

The poor solubility of many nucleosides and nucleobases in aqueous solution demands harsh reaction conditions (base, heat, cosolvent) in nucleoside phosphorylase‐catalyzed processes to facilitate substrate loading beyond the low millimolar range. This, in turn, requires enzymes that can withstand these conditions. Herein, we report that the pyrimidine nucleoside phosphorylase from *Thermus thermophilus* is active over an exceptionally broad pH (4–10), temperature (up to 100 °C) and cosolvent space (up to 80 % (*v*/*v*) nonaqueous medium), and displays tremendous stability under harsh reaction conditions with predicted total turnover numbers of more than 10^6^ for various pyrimidine nucleosides. However, its use as a biocatalyst for preparative applications is critically limited due to its inhibition by nucleobases at low concentrations, which is unprecedented among nonspecific pyrimidine nucleoside phosphorylases.

## Graphical abstract

**What**? The pyrimidine nucleoside phosphorylase from *Thermus thermophilus* is an outlier among its peers. The enzyme is extremely stable and performs remarkably well in near‐boiling, cosolvent‐heavy media, but an apparent inhibition by nucleobases renders its performance in preparative applications rather subpar.

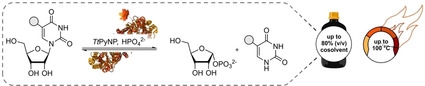

Nucleoside phosphorylases are useful biocatalysts for the synthesis of pentose‐1‐phosphates and nucleoside analogues.[^1^, ^2^, ^3^, ^4^, ^5^, ^6^, ^7^, ^8^, ^9^, ^10^, ^11^] These enzymes catalyze the reversible phosphorolysis of nucleosides to the corresponding pentose‐1‐phosphates and nucleobases (Scheme 1) and can be employed in the reverse reaction for glycosylation and transglycosylation reactions to furnish nucleosides of interest directly from free nucleobases. The current primary bottleneck for the efficient application of these enzymes for synthetic purposes is the low water solubility of many nucleosides and nucleobases, restricting the substrate loading to the low‐millimolar range.^[12]^ Increased substrate solubility can be achieved through the use of harsh reaction conditions (base, heat, cosolvent) and, consequently, thermostable nucleoside phosphorylases have attracted particular interest due to their stability and activity under these conditions.[^1^, ^2^, ^4^] Recent work from our group has demonstrated that some of these enzymes can be employed reliably at temperatures of up to 70 °C and over a broad pH range up to at least pH 9, which creates an exceptionally large working space and allows adjustments of the reaction conditions to suit the substrate(s).[^7^, ^13^, ^14^] Further benefits of these thermostable enzymes, including their long shelf life and easy purification via heat treatment of crude extracts, make these catalysts attractive for various applications.

**Scheme 1:**
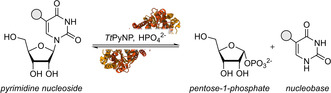
Phosphorolysis of pyrimidine nucleosides catalyzed, e. g., by *T. thermophilus* pyrimidine nucleoside phosphorylase (*Tt*PyNP, PDB ID: 2DSJ).

The pyrimidine nucleoside phosphorylase from *Thermus thermophilus* (*Tt*PyNP, Scheme 1) has been reported to be active at up to 100 °C in aqueous media and to display some activity in reaction mixtures containing high concentrations of organic solvents like DMSO and DMF.^[15]^ Therefore, we hypothesized that such a hyper‐thermostable enzyme would withstand harsh conditions such as near‐boiling cosolvent‐heavy media and enable greatly increased substrate loading compared to commonly performed nucleoside transglycosylations.[^8^, ^9^, ^16^] This prompted us to investigate *Tt*PyNP in more detail for applications in nucleoside synthesis and probe the limits of its tolerance to harsh conditions. In this communication, we report that *Tt*PyNP is active and stable over a vast working space regarding temperature, pH and cosolvents and accepts a range of substituted pyrimidine nucleosides. In the course of our work we were surprised to discover that *Tt*PyNP is inhibited by nucleobases even at low concentrations, which is atypical for pyrimidine nucleoside phosphorylases. Hence, *Tt*PyNP is a suboptimal candidate for industrial applications where high substrate loading is a prerequisite.

Following heterologous expression of the enzyme in *Escherichia coli* and purification by heat treatment and affinity chromatography, we explored the working space of *Tt*PyNP using the phosphorolysis of uridine (**1 a**) as a model reaction (Figure 1A). Since this reaction is under tight thermodynamic control (*K*=0.2 at 60 °C and pH 9),^[7]^ we applied an excess of phosphate in these experiments to drive the reaction in the phosphorolysis direction. *Tt*PyNP displayed phosphorolytic activity from pH 4 to 10 (Figure 1B), with a clear preference for acidic conditions as the observed rate constants *k*_obs_ differed by more than a factor of five between pH 4 and 10. This observation is somewhat counterintuitive since i) pentose‐1‐phosphates are prone to hydrolysis under acidic conditions,[^2^, ^17^] ii) an intracellular pH in the range of 7 would suggest a preference for neutral conditions and iii) other pyrimidine nucleoside phosphorylases are inactive at pH values below 6.^[18]^ In accordance with previous reports,[^13^, ^15^] *Tt*PyNP was active at temperatures of up to 100 °C (Figure 1C), with the temperature dependence of *k*_obs_ (in contrast to previous reports)^[15]^ following the trends predicted by conventional transition state theory as described by the Eyring equation.^[19]^ These results indicated that the working space of *Tt*PyNP covers most of the pH and temperature range accessible in water and, given the activity of the enzyme, we anticipated that its overall performance would only be limited by its stability under the applied reaction conditions.

**Figure 1:**
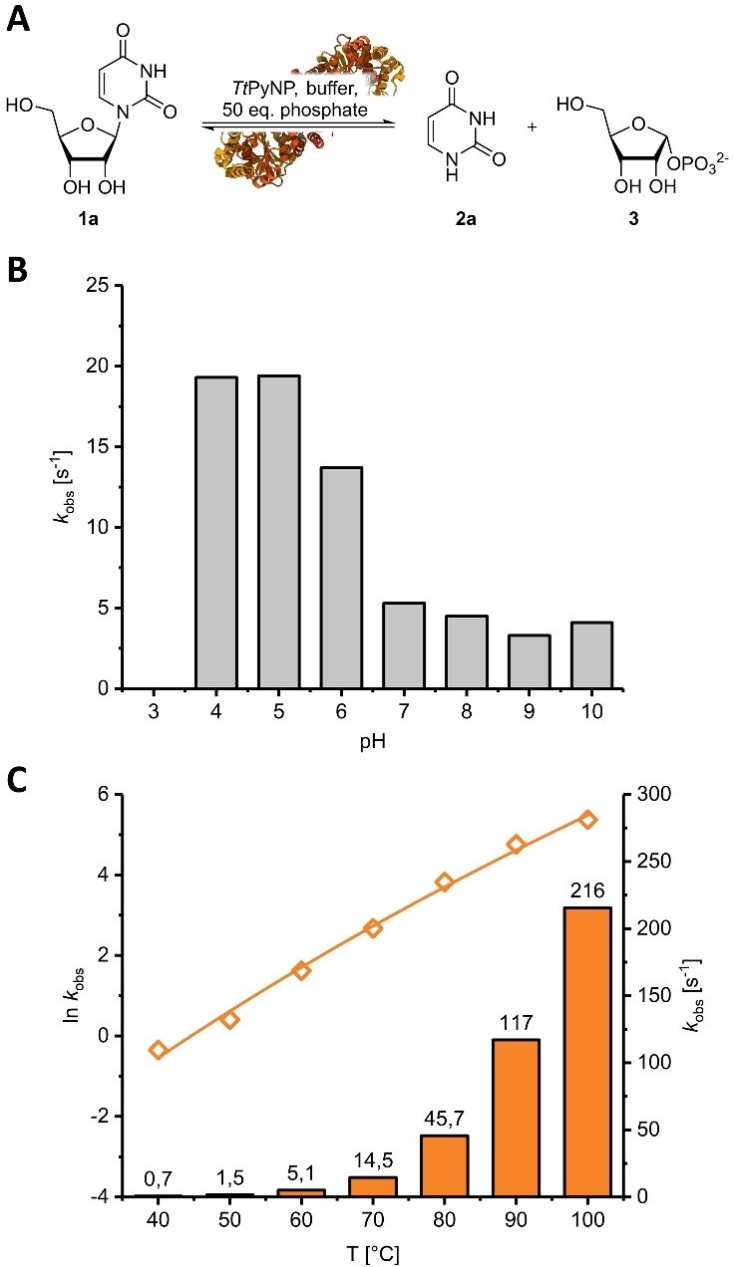
Working space of *Tt*PyNP. A) The phosphorolysis of uridine (**1 a**) was employed to investigate B) the pH and C) temperature dependence of the phosphorolytic activity as quantified by the observed rate constant *k*_obs_. To assay across the pH space, reactions were performed with 1 mM **1 a**, 50 mM potassium phosphate, 2 or 8 μg mL^−1^ *Tt*PyNP at 60 °C in a buffer mix consisting of 5 mM citrate, 10 mM MOPS and 20 mM glycine (all final concentration; adjusted to the respective pH value at 25 °C, not equated for ionic strength) in a final volume of 500 μL. To assay across the temperature space, reactions were carried out with 1 mM **1 a**, 50 mM potassium phosphate and 0.06–20 μg mL^−1^ *Tt*PyNP in 50 mM glycine/NaOH buffer at pH 9 and the indicated temperature. Data shown represent the average of two experiments. Error bars are too small to see and were omitted for clarity. Fit results for (C) are available in the Supporting Information. Please see the Supporting Information for more experimental details and the externally hosted Supporting Information for raw data and calculations.^[20]^

Next, we turned our attention to the activity of *Tt*PyNP in the presence of organic cosolvents. Previous work with this enzyme indicated that some activity is retained in mixtures containing 50 % (*v*/*v*) DMSO or DMF.^[15]^ These two solvents fall into the short list of common organic solvents that meet the prerequisites to be considered as cosolvents for high temperature biocatalytic reactions involving nucleosides: i) a boiling point >100 °C, ii) water miscibility^[21]^ and iii) resistance to hydrolysis. These prerequisites readily disqualify commonly used alcohols such as methanol, ethanol or isopropanol,^[22]^ as well as aromatics, ethers, hydrocarbons and esters. Considering that DMF is toxic, highly harmful to the environment^[23]^ and rather detrimental to the activity of *Tt*PyNP (Figure S1), we selected DMSO and ethylene glycol as promising cosolvents and interrogated *Tt*PyNP's activity at different concentrations of these solvents, again employing the phosphorolysis of **1 a** as a model reaction. *Tt*PyNP displayed activity at up to 60 % (*v*/*v*) DMSO and 80 % (*v*/*v*) ethylene glycol, with more cosolvent being tolerated at 80 than at 90 °C (Figure 2). Interestingly, moderate amounts of either cosolvent (i. e. 20–40 %, *v*/*v*) promoted higher activities than observed in purely aqueous solution, presumably due to decreased enzymatic flexibility and a higher affinity for both substrates in a slightly less polar environment.[^24^, ^25^]

**Figure 2:**
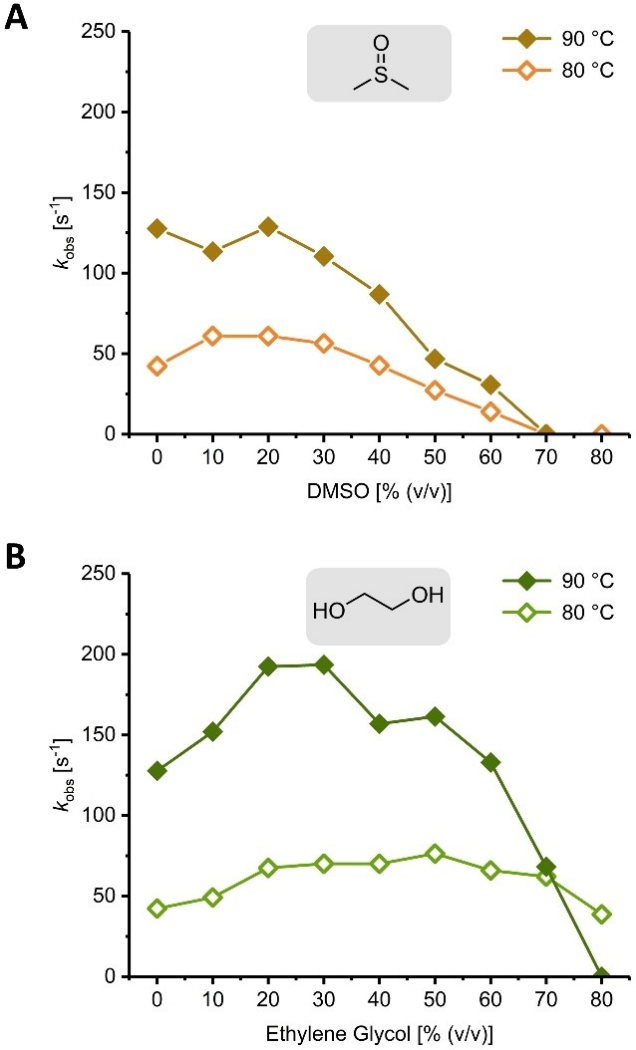
Phosphorolytic activity of *Tt*PyNP in the presence of the organic cosolvents dimethyl sulfoxide (DMSO, A) and ethylene glycol (B). Reactions were run with 1 mM **1 a**, 20 mM potassium phosphate and 0.12 or 0.40 μg mL^−1^ *Tt*PyNP in 20 mM glycine/NaOH buffer pH 9 containing the indicated amount of cosolvent at the indicated temperature. Data shown represent the average of two experiments. Error bars are too small to see and were omitted for clarity. Please see the Supporting Information for more experimental details and the externally hosted Supporting Information for raw data and calculations.^[20]^

Having established the activity of *Tt*PyNP at high temperatures and with considerable amounts of cosolvent, we were interested in the half‐life and total turnovers of the enzyme to evaluate if these harsh conditions were feasible for any reactions that exceeded the short reaction times of activity assays. To determine the half‐life *t*_1/2_ and predicted total turnover number (pTTN) of *Tt*PyNP, we incubated the enzyme in buffered solution with phosphate, determined its residual activity after various incubation times and approximated the incubation time‐dependent decrease in activity as a first‐order exponential decay. Despite its remarkable activity at 100 °C and pH 9 in purely aqueous solution (Figure 1B), the half‐life of *Tt*PyNP under these conditions was only 2.2 min, corresponding to a pTTN of only around 28 000 (Table 1, entry 1). At 90 and 80 °C, we found half‐lives of 100 min and more than 19 h, respectively, with the pTTNs increasing accordingly to well above 10^6^, despite the lower turnover rates at these temperatures (Table 1, entries 2 and 3). These experiments demonstrated that denaturation‐driven loss of activity proceeds rapidly near the boiling point of water. However, slightly lower temperatures facilitated increased stability and enzymatic activity, as reflected by the predicted total turnovers. As transformations at 80–90 °C appeared feasible from a stability standpoint, we then assessed the stability of *Tt*PyNP in the presence of DMSO and ethylene glycol, using the same experimental set‐up. Although both solvents permitted excellent activity of *Tt*PyNP at 90 °C, the half‐life of the enzyme did not surpass 30 min at 40 % (*v*/*v*) of either solvent at this temperature, reflected by pTTNs below 200 000 (Figure 3). Nonetheless, a modest decrease in temperature to 80 °C resulted in much higher half‐lives and pTTNs. For example, *Tt*PyNP had a half‐life of 4.4 h at 80 °C and 60 % (*v*/*v*) ethylene glycol, corresponding to around 1 500 000 predicted total turnovers (Figure 3B). Even DMSO was tolerated reasonably well, with *Tt*PyNP achieving a pTTN of 3 100 000 at 80 °C and 30 % (*v*/*v*) DMSO (Figure 3A). Taken together, these data demonstrate that *Tt*PyNP performs favorably at up to 80 °C in media with up to around 50 % (*v*/*v*) of DMSO or ethylene glycol.

**Table 1:** Activity and stability of *Tt*PyNP in aqueous solution.

*T* [°C]	*k*_obs_ [s^−1^]^[a]^	*t*_1/2_^[b]^	pTTN [10^6^]^[c]^
100	215.6	2.2 min	0.028
90	117.1	100.2 min	1.296
80	45.7	19.5 h	15.139

[a] Determined as initial rate with **1 a** at pH 9 and the respective temperature as shown in Figure 1C. [b] Determined from incubation experiments from the incubation time‐dependent decay of enzymatic activity (initial rate) at pH 9 and the respective temperature (please see the externally hosted Supporting Information for all raw and calculated data).^[20]^ The activity assay was performed at 60 °C to ensure enzyme stability during the assay. [c] Determined via multiplication of *k*_obs_ and the mean catalyst lifetime τ as detailed in the Supporting Information.

**Figure 3:**
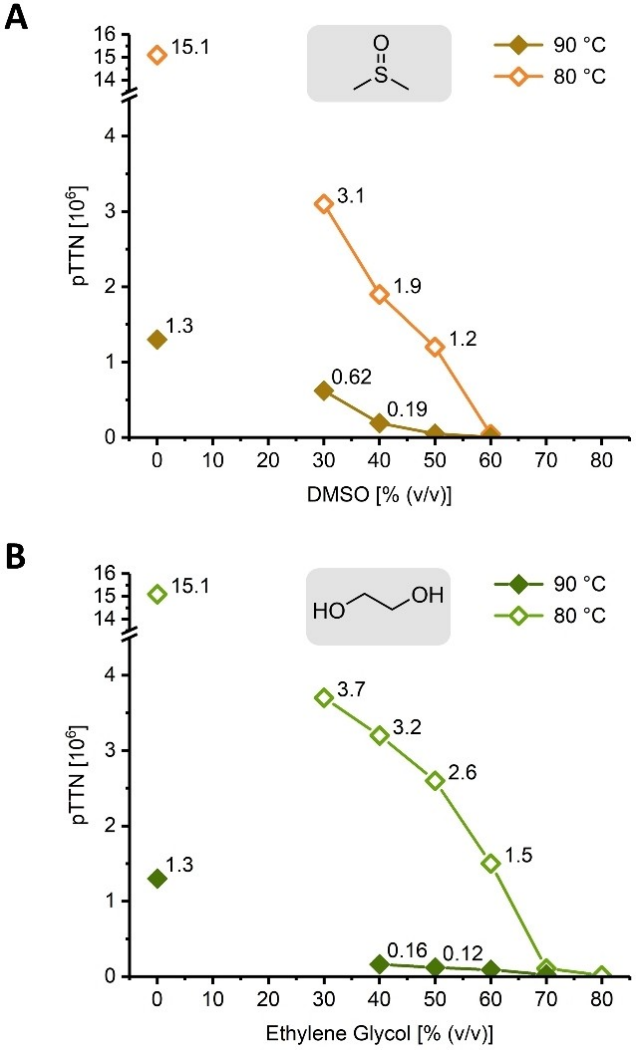
Performance of *Tt*PyNP assessed by the predicted total turnover number (pTTN) of the substrate **1 a** in hot cosolvent‐heavy reaction mixtures. The half‐life of *Tt*PyNP was determined by incubating the enzyme at concentrations of 8.4–12.5 μg mL^−1^ in 20 mM potassium phosphate and 20 mM glycine/NaOH buffer pH 9 and the indicated concentrations of A) DMSO or B) ethylene glycol at the indicated temperatures in a total volume of 220 μL in a PCR tube, and measuring the residual activity through phosphorolysis of the model substrate **1 a** at timely intervals (assay conditions of 1 mM **1 a**, 20 mM potassium phosphate and 2.5–3.75 μg mL^−1^ *Tt*PyNP in 20 mM glycine buffer pH 9 at 60 °C). Fitting the incubation time‐dependent decrease in activity as a first‐order exponential decay yielded the half‐life under the respective conditions, which was used to calculate pTTN via the initial rate constant under these conditions (Figure 2). Please see the externally hosted Supporting Information for all raw and calculated data as well as tabulated half‐lives and predicted total turnover numbers.^[20]^

Next, we probed the substrate scope of this enzyme by subjecting a series of substituted pyrimidine nucleosides to phosphorolysis. Previous work had demonstrated that **1 a**, its 2’‐deoxy analogue **1 b** and thymidine (**1 d**) are converted by *Tt*PyNP to obtain the corresponding sugar phosphate and nucleobase.[^13^, ^15^] Our results corroborate and extend these reports by showing that substitutions in the 5‐position at the nucleobase, such as aliphatic residues or halogens, are generally well tolerated without any loss of activity compared to the native substrates **1 a** and **1 d** (Table 2). Comparable or slightly higher levels of activity were obtained with 2’‐deoxy nucleosides, which agrees well with the broad substrate spectrum typically observed for non‐specific pyrimidine nucleoside phosphorylases.[^7^, ^8^, ^9^] Given all the above results, *Tt*PyNP indeed seemed useful as a catalyst for the (reversible) phosphorolysis of various pyrimidine nucleosides as it displays a reasonably broad substrate scope and remarkable stability under harsh conditions including high temperature and cosolvent content.

**Table 2:** Phosphorolysis of 5‐substituted pyrimidine nucleosides by *Tt*PyNP.^[a]^

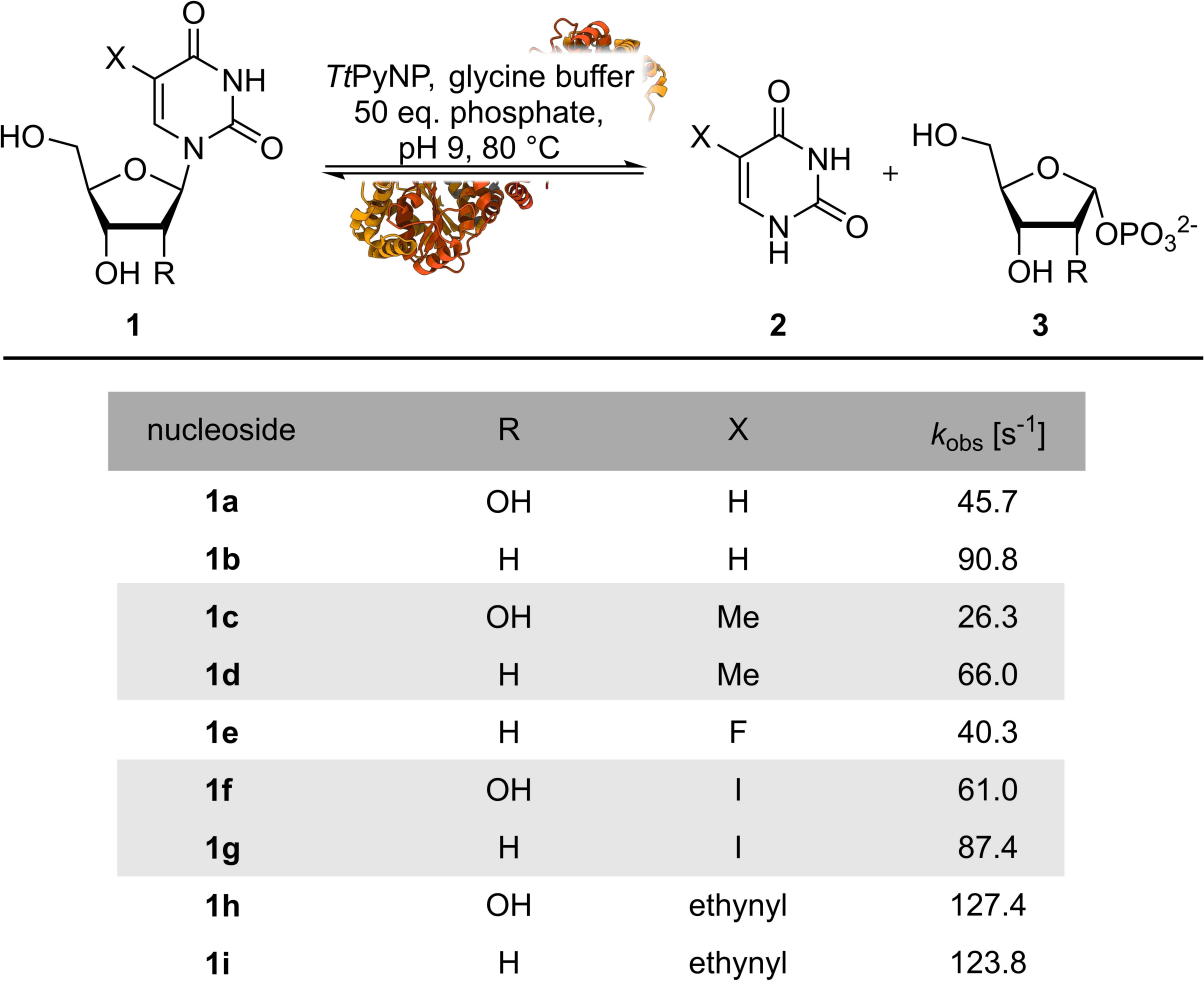

[a] Reactions were performed with 1 mM nucleoside (**1 a**–**1 i**), 50 mM potassium phosphate and 0.25 μg mL^−1^ *Tt*PyNP in 50 mM glycine/NaOH buffer at pH 9 and 80 °C. Data shown represent the average of two experiments. Standard deviations were all 5 % or below and were omitted for clarity. Please see the Supporting Information for more experimental details and the externally hosted Supporting Information for raw data and calculations.^[20]^

However, despite *Tt*PyNP's excellent activity and stability, its use as a biocatalyst in synthetic applications is limited due to an apparent inhibition by nucleobases. We discovered this phenomenon when we attempted to determine the Michaelis–Menten constant *K*_M_ for the substrate **1 a** using concentrations of up to 50 mM (Figure S2). Previous work had reported *K*_M_ values in the range of 0.2–1.0 mM at 80 °C and pH 7 (for **1 a** and **1 d** in phosphate buffer)[^13^, ^15^] which compares well with other pyrimidine nucleoside phosphorylases.[^26^, ^27^, ^28^] However, our data at 40 °C and pH 9 suggested a strong inhibition behavior which did not appear to resemble any Michaelis–Menten‐type kinetics (Figure S2). Follow‐up experiments with **1 a** at 80 °C and pH 7 revealed that, indeed, *Tt*PyNP appeared to follow Michaelis‐Menten‐like kinetics for nucleoside concentrations up to 1–2 mM (under saturating phosphate concentrations for which typical Michaelis–Menten behavior was observed; Figure S4). In contrast, the observed rate constants of phosphorolysis at higher nucleoside concentrations suggested a possible substrate inhibition by nucleosides with an apparent inhibitory constant *K*_i_ within the same order of magnitude as *K*_M_. However, the non‐linearity of the conversion over time at higher substrate concentrations under quasi‐steady state conditions (Figure S5) provided some evidence that *Tt*PyNP might instead be inhibited by one of the products. Indeed, non‐zero initial concentrations of the nucleobase **2 a** caused a drastic decrease of the *k*_obs_ of phosphorolysis, while the sugar phosphate **3** had no effect (Figure 4A). Due to this strong inhibition of *Tt*PyNP by **2 a**, we were unable to determine the *K*_i_ from Michaelis−Menten plots of the phosphorolysis reaction. Therefore, we attempted to approximate the *K*_i_ by measuring the *K*_M_ for **2 a** in the reverse reaction (glycosylation with **3**). However, instead of saturation‐type kinetics we only observed a significant decrease of the *k*_obs_ of glycosylation with increasing concentrations of **2 a**, indicating that this nucleobase also inhibits its own glycosylation (Figure 4B). To exclude any of our assay conditions causing these effects, we determined the *K*_M_ for **1 a** of the corresponding enzyme from *Geobacillus thermoglucosidasius* (*Gt*PyNP) and observed classic Michaelis–Menten behavior with no evidence of inhibition up to 50 mM **1 a** in agreement with the available literature (Figure S3).^[13]^ Interestingly, similar inhibition effects of *Tt*PyNP were also observed, to varying degrees, with the nucleosides **1 d** and **1 f** (Figure S6), revealing that inhibition of this enzyme by nucleobases is not limited to **2 a**. Together, these results present evidence that *Tt*PyNP is inhibited by nucleobases through a yet unknown mechanism. The reason(s) for this apparent product inhibition (or substrate inhibition, depending on the direction) are unclear to date as *Tt*PyNP shares high structural^[29]^ and sequence identity (see Figure 1 in ref. [13]) to other thymidine and pyrimidine nucleoside phosphorylases concerning the active site residues and overall protein structure. Although substrate inhibition is known for uridine phosphorylases,[^28^, ^30^] this is, to the best of our knowledge, the first reported example of a pyrimidine nucleoside phosphorylase being competitively substrate/product‐inhibited. However, we doubt that this inhibition of *Tt*PyNP holds any physiological significance, as the intracellular concentrations of nucleosides and their bases are typically in the low‐micromolar range, which is more than two orders of magnitude lower than the concentrations necessary to effect significant inhibition of *Tt*PyNP. In any case, this clearly makes *Tt*PyNP a rather suboptimal candidate for preparative purposes. In order to achieve satisfactory product titers, substrate concentrations of at least 100 mM typically need to be applied.[^12^, ^31^, ^32^] Our characterization revealed that *Tt*PyNP is severely inhibited by nucleobases at concentrations upwards of 0.5 mM, which limits its reactivity both in the phosphorolysis and glycosylation direction and renders its performance in potential industrial applications subpar.

**Figure 4:**
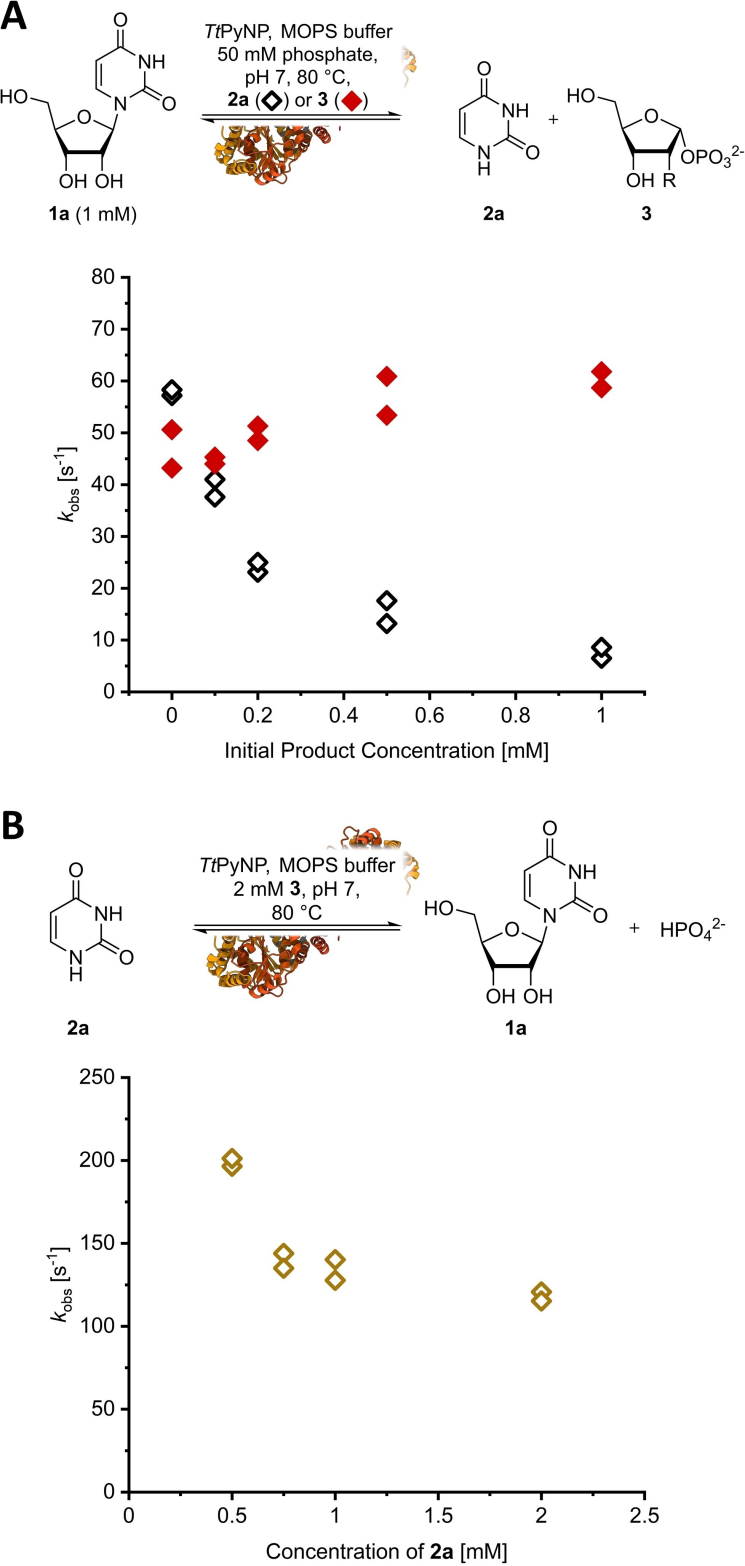
Inhibition of *Tt*PyNP by substrates and products. A) *Tt*PyNP is inhibited in the phosphorolysis direction by increasing concentrations of the nucleobase **2 a**, but not by the sugar phosphate **3**. B) The reverse reaction is also inhibited by increasing concentrations of **2 a**. Reactions in (A) were carried out with 1 mM **1 a**, 50 mM potassium phosphate and 0.33 mL^−1^ *Tt*PyNP in 50 mM MOPS/NaOH buffer pH 7 at 80 °C with the indicated concentration of either product (**2 a** or **3**) in a total volume of 200 μL. Reactions in (B) were performed with 2 mM **3** and 0.17 μg mL^−1^ *Tt*PyNP in 50 mM MOPS/NaOH buffer pH 7 at 80 °C with the indicated concentration of **2 a** in a total volume of 200 μL. For each condition, reactions were carried out in duplicate and all data points are shown. Raw data and calculations are freely available from an external online repository.^[20]^

In conclusion, we have characterized the hyper‐thermostable pyrimidine nucleoside phosphorylase from *Thermus thermophilus* and revealed its exceptional working space, broad substrate scope as well as excellent stability and tolerance to organic cosolvents. However, its inhibition by nucleobases even at low concentrations discourages its use in synthetic applications. These observations make *Tt*PyNP an outlier among pyrimidine nucleoside phosphorylases, displaying unmatched stability and a rare example of substrate/product inhibition.

## Experimental Section

Enzymatic reaction mixtures were prepared from stock solutions of nucleoside, potassium phosphate and buffer and started via the addition of enzyme. Reaction conditions were applied as stated in the figure captions and the Supporting Information. Reaction monitoring was performed as described previously.[^7^, ^33^, ^34^] Typically, samples of 50 μL were withdrawn from reaction mixtures containing 1 mM UV‐active compounds and pipetted into 450 μL 100 mM aqueous NaOH to stop the reaction. To record the UV spectra of these alkaline samples, 200 μL of the quenched samples were transferred to a UV/Vis‐transparent 96‐well plate (UV star, GreinerBioOne, Kremsmünster, Austria) and UV absorption spectra were recorded from 250 to 350 nm in steps of 1 nm with a high‐throughput plate reader (BioTek Instruments, Winooski, USA). The obtained experimental UV spectra were then deconvoluted via spectral unmixing using suitable reference spectra of the nucleoside and nucleobase to derive the respective degree of conversion. All raw data presented in this report, along with metadata and calculations are freely available from an external online repository.^[20]^ Likewise, reference spectra and software for spectral unmixing are available from the same repository[^35^, ^36^] and described in previous works.[^33^, ^34^]

## Supporting information

As a service to our authors and readers, this journal provides supporting information supplied by the authors. Such materials are peer reviewed and may be re‐organized for online delivery, but are not copy‐edited or typeset. Technical support issues arising from supporting information (other than missing files) should be addressed to the authors.

Supplementary
