# Supplementary material for: The Peculiar Case of the Hyper‐thermostable Pyrimidine Nucleoside Phosphorylase from Thermus thermophilus
Source: Chembiochem. 2021 Jan 26;22(8):1385–90. doi: 10.1002/cbic.202000679 (PMC8247850; doi:10.1002/cbic.202000679)
Supplement: Supplementary file 1 — Supplementary [file CBIC-22-1385-s001.pdf]

ChemBioChem

Supporting Information

The Peculiar Case of the Hyper-thermostable Pyrimidine Nucleoside Phosphorylase from *Thermus thermophilus***

Felix Kaspar,* Peter Neubauer, and Anke Kurreck*

Author Contributions

F.K. Conceptualization:Equal; Data curation:Lead; Formal analysis:Lead; Investigation:Lead; Methodology:Lead; Project administration:Equal; Visualization:Lead; Writing – original draft:Lead; Writing – review & editing:Equal
P.N. Conceptualization:Equal; Funding acquisition:Equal; Resources:Lead; Supervision:Supporting; Writing – review & editing:Equal
A.K. Conceptualization:Equal; Funding acquisition:Lead; Project administration:Equal; Resources:Equal; Supervision:Lead; Writing – review & editing:Equal

Author contributions	1
Data availability	1
General remarks	1
Protein sequence of *Tt*PyNP	2
Experimental	2
Supplementary items	8
Supplementary references	13

Author contributions (with definitions as recommended by Brand *et al.*[1])

Conceptualization, F.K., P.N. and A.K.; Data curation, F.K.; Formal analysis, F.K.; Funding acquisition, P.N. and A.K.; Investigation, F.K.; Methodology, F.K.; Project administration, F.K. and A.K.; Resources, P.N. and A.K.; Software, - ; Supervision, P.N. and A.K.; Validation, - ; Visualization, F.K.; Writing—original draft, F.K.; Writing—review & editing, F.K., P.N. and A.K.

Data availability

All data depicted visually in the items in the main text (Figures 1−4, Tables 1 and Scheme 2) as well as in the Supplementary Information (Figures S1−6, see below) is available as tabulated data from the externally hosted Supporting Information at zenodo.org.[2]

General remarks

All chemicals used in this study were of analytical grade or higher and purchased from Sigma Aldrich (Steinheim, Germany), Carbosynth (Berkshire, UK), Carl Roth (Karlsruhe, Germany), TCI Deutschland (Eschborn, Germany) or VWR (Darmstadt, Germany) and used without prior purification. Water deionized to 18.2 MΩ·cm with a Werner water purification system was used for the preparation of all enzymatic reactions as well as purification and storage buffers. For the preparation of NaOH solutions for quenching, deionized water was used.

Protein sequence of *Tt*PyNP

MRGSHHHHHHGSNPVAFIREKREGKKHRREDLEAFLLGYLRDEVPDYQVSAWLMAAFLRGLDPEETLWLTETMA
RSGKVLDLSGLPHPVDKHSSGGVGDKVSLVVGPILAASGCTFAKMSGRGLAHTGGTIDKLESVPGWRGEMTEAEFL
ERARRVGLVIAAQSPDLAPLDGKLYALRDVTATVESVPLIASSIMSKKLAAGARSIVLDVKVGRGAFMKTLEEARLLA
KTMVAIGQGAGRRVRALLTSMEAPLGRAVGNAIEVREAIEALKGEGPGDLLEVALALAEEALRLEGLDPALARKALE
GGAALEKFRAFLEAQGGDPRAVEDFSLLPLAEEHPLRAEREGVVREVDAYKVGLAVLALGGGRKRKGEPIDHGVGV
YLLKKPGDRVERGEALALVYHRRRGLEEALGHLREAYALGEEAHPAPLVLEAI-

Experimental

Protein expression was performed as described recently.[3,4] Briefly, all enzymes were heterologously expressed in *E. coli* as His_6-tagged proteins through IPTG-induced overexpression. Purification was achieved through cell disruption, heat treatment of the crude extract (80 °C for *Tt*PyNP, 60 °C for *Gt*PyNP, both for 30 min) and Ni-NTA affinity chromatography. Proteins were eluted with buffer containing 250 mM imidazole, 50 mM sodium phosphate, 300 mM NaCl (pH 8) and stored as stock solutions at −20 °C in 50% (v/v) glycerol. Typical protein stock concentrations were around 1 g L^{-1} (calculated with 1 AU cm^{-1} at 280 nm being equal to a protein concentration of 1 g L^{-1}). Under these storage conditions, no decay of activity could be detected over the course of more than 6 months.

Enzymatic reactions were performed in 1.5 mL Eppendorf tubes and prepared from stock solutions of nucleoside, potassium phosphate and buffer (optionally with additional reaction components, such as organic cosolvents). Typical reaction volumes were 200 or 500 µL, depending on the experiment and substrate concentration. Reactions were preheated to the respective temperature for 30 s to 1 min and initiated by the addition of 5−10 µL suitably diluted enzyme stock solution (predilution in 2 mM potassium phosphate buffer, pH 7). Samples were withdrawn at timely intervals, typically after 1, 2 and 3 min after reaction initiation or as detailed in the metadata files freely available online.[2] Unless stated otherwise, all reactions were performed in duplicate.

Reaction monitoring was achieved via spectral unmixing. From live reactions, samples were withdrawn and quenched in aqueous NaOH as described previously.[4,5] For the substrates **1a**−**1d**, samples were quenched in 100 mM NaOH and for **1e**−**1i** in 200 mM NaOH, as detailed in our previous work.[5] Sample dilution factor was adjusted to reach final concentrations of 100−150 µM UV-active reaction components (please note that the exact concentration is not relevant here since spectral unmixing only takes spectral shape and not absolute intensity into account). Of the diluted alkaline sample, 200 µL were transferred to UV/Vis-transparent 96-well plates (UV star, GreinerBioOne, Kremsmünster, Austria) for analysis. UV absorption spectra were recorded from 250−350 nm with a BioTek PowerWave HT platereader and subjected to spectral unmixing using analogously obtained reference spectra.[2] Reference spectra used in this study are freely available in the externally hosted

Supplementary Information[2] and can, alternatively, be obtained from the Supplementary Information of previous publications.[6] The degree of conversion was determined directly from the spectra fit which considers the UV-active substrate and product in relation to one another.[4] For activity determination, only sampling points showing 3−10% conversion of the nucleoside substrate were considered. This lower bound was set due to the inherent inaccuracy of the UV-based method employed (roughly ±0.3 percentage points, due to the inherent error in spectral acquisition, as described in the original publication)[4] and the upper bound was applied as recommended by Cornish-Bowden[7] for equilibrium reactions. All datapoints outside this window were not included for calculation of activity and marked accordingly in the Supplementary Information.[2] Datapoints that displayed baseline shifts or other spectral anomalies were also excluded from consideration. Background correction was performed as described recently.[5] Experimental spectra were fitted either across the entire spectrum or over the information-rich shoulder region of pyrimidine nucleosides/nucleobases, as appropriate for the analysis. All background corrections and the corresponding datafiles are detailed in the metadata files in the externally hosted supplementary information.[2]

Enzymatic activity was determined by linear approximation of the conversion over time with a forced intercept at the origin. All raw data and the datapoints considered for calculation are freely available online with outliers and excluded datapoints clearly marked.[2] The observed rate constant k_{obs} was obtained by considering the degree of conversion (mol per second) per mol enzyme applied, using the molar extinction coefficient of *Tt*PyNP of 26.930 cm^{-1} M^{-1} as predicted by Protparam[8] (i.e. the stock solution of 1 g L^{-1} had a concentration of 37.1 µM).

The activity of TtPyNP between pH 3 and 10 was determined using reaction mixtures of 1 mM **1a**, 50 mM potassium phosphate and 8 µg mL^{-1} *Tt*PyNP (2 µg mL^{-1} for pH 4−6) at 60 °C in a buffer mix consisting of 5 mM citrate, 10 mM MOPS and 20 mM glycine (all final concentration; adjusted to the respective pH value with NaOH and HCl solutions, not equated for ionic strength) in a final volume of 500 µL. Samples of 50 µL were withdrawn, quenched and analyzed after 1, 2 and 3 min as described above.

The activity of TtPyNP from 40−100 °C was determined using reaction mixtures of 1 mM **1a** and 50 mM potassium phosphate in 50 mM glycine/NaOH buffer at pH 9 and the indicated temperature in a total volume of 500 µL. Depending on the temperature (and, therefore, on rate of phosphorolysis), 0.06−20 µg mL^{-1} *Tt*PyNP were used (Table S1), to permit sampling of all reactions within the same time domain. From all reactions, samples of 50 µL were withdrawn after 1, 2 and 3 min, quenched and analyzed as described above. The obtained data were fit to the Eyring equation[9]

$$k_{obs} = \frac{\kappa k_b T}{h} \exp\left(-\frac{\Delta_R H^{\ddagger} - T\Delta_R S^{\ddagger}}{RT}\right) \quad (1)$$

where k_{obs} is the observed rate constant [s^{-1}], κ is the transmission coefficient (herein assumed to be unity), k_b is the Boltzmann constant ($1.38 \cdot 10^{-23}$ J K^{-1}), T is the temperature [K], h is the Planck constant ($6.626 \cdot 10^{-34}$ J s), R is the universal gas constant (8.314 J mol^{-1} K^{-1}), $\Delta_R H^{\ddagger}$ the transition state enthalpy [J mol^{-1}] and $\Delta_R S^{\ddagger}$ is the transition state entropy [J mol^{-1} K^{-1}].
The fit of the experimental data shown in Figure 1C yielded $\Delta_R H^{\ddagger}$ = 94.68 ± 3.06 kJ mol^{-1} and $\Delta_R S^{\ddagger}$ = 27.04 ± 1.08 J mol^{-1} K^{-1} (R^2 = 0.994).

Table S1. Enzyme amounts used for kinetics assays at different temperatures.

T [°C]	*Tt*PyNP concentration [µg mL^{-1}]
40	20
50	10
60	5
70	2
80	0.4
90	0.16
100	0.06

The activity of TtPyNP in reaction mixtures with cosolvent was determined by assaying for the initial rate of phosphorolysis of **1a**. Reactions with 1 mM **1a**, 20 mM potassium phosphate, 0.12–0.40 µg mL^{-1} *Tt*PyNP (0.12 µg mL^{-1} for 90 °C and 0.40 µg mL^{-1} for 80 °C) in 20 mM glycine/NaOH buffer pH 9 containing the indicated amount of cosolvent in a total volume of 500 µL were performed with samples taken after 1, 2 and 3 min. Quenching and data analysis was carried out as described above and data were fit as detailed in the metadata files available online.[2]

The half-life of TtPyNP under different conditions was determined by incubation of *Tt*PyNP (8.4–12.5 µg mL^{-1}) in 20 mM potassium phosphate and 20 mM glycine/NaOH buffer pH 9 at the indicated temperatures in a total volume of 220 µL in a PCR tube (please note that the PCR tube was chosen intentionally for incubation since it ensures homogenous heating of the entire mixture without any sample cooling or evaporation). The tubes were incubated in a PCR cycler with lid heating for various timespans at a constant temperature as indicated. The lid temperature was set to 10 °C above the incubation temperature to prevent sample condensation at the lid. At certain timepoints, tubes were removed from the PCR cycler and immediately cooled on ice until activity determination. To assay for (residual) enzymatic activity, 150 µL of the incubated enzyme mixture were used to start a reaction consisting of 1 mM **1a**, 20 mM potassium phosphate, 20 mM glycine/NaOH buffer pH 9 at 60 °C in a total volume of 500 µL (i.e. 150 µL enzyme mixture were added to 350 µL nucleoside mixture with both

mixtures containing the same phosphate and buffer concentrations and a final total concentration of 1 mM **1a** and 2.50–3.75 µg mL^{-1} *Tt*PyNP). The assay was performed at 60 °C to ensure enzymatic activity during the activity assay without denaturation during the reaction. From these reactions, samples of 50 µL were withdrawn after 1, 2 and 4 min (or as indicated in the metadata file), quenched and analyzed as described above. Reactions containing organic solvent were performed analogously with the incubation mixture and the assay mixture containing the respective concentration of cosolvent. All data regarding residual activity were compared to the activity without any incubation (both aqueous and cosolvent incubations, i.e. since initial rate varied with cosolvent content, these data were referenced to the initial rate under exactly these conditions). To obtain the half-life of *Tt*PyNP, activity data were fit to the first-order exponential decay function

$$k_{obs}(t) = k_{obs,0}\, e^{-\frac{t}{\tau}} \qquad (2)$$

where $k_{obs}(t)$ is the rate constant [s^{-1}] observed after an incubation time t [min] (or [h]), $k_{obs,0}$ is the observed rate constant prior to incubation [s^{-1}] and τ is the mean catalyst lifetime [min] (or [h]) from which the half-life $t_{1/2}$ [min] (or [h]) can be obtained via

$$t_{1/2} = \tau\, ln(2) \qquad (3)$$

with definitions from above. Please see Table S2 for an overview of all incubated enzyme amounts and incubation times for each condition. All raw and transformed data can be obtained free of charge from an external online repository.[2]

The predicted total turnovers pTTN were calculated as

$$\mathrm{pTTN} = \tau\, k_{obs,0}(T) \qquad (4)$$

with $k_{obs,0}(T)$ being the observed initial rate constant at the given temperature (as shown in Figures 1C, 2A and 2B), and definitions from above.

Please see the externally hosted Supporting Information for all raw data and fit results.[2]

Table S2. Incubation conditions and sampling times for half-life determination of *Tt*PyNP under harsh conditions.

Incubation condition			
T [°C]	cosolvent, % (v/v)	enzyme incubated [µg]	sampling times [min]
100	-	5	3, 6, 9
90	-	5	80, 160, 240
80	-	5	80, 1170, 2250
90	DMSO, 60	7.5	2, 5, 10
90	DMSO, 50	7.5	20, 40, 60
90	DMSO, 40	5	20, 40, 60
90	DMSO, 30	5	20, 40, 60
80	DMSO, 60	7.5	17, 34, 92
80	DMSO, 50	7.5	137, 1174, 1601
80	DMSO, 40	5	1174, 1601
80	DMSO, 30	5	1174, 1601
90	ethylene glycol, 70	5	2, 5, 10
90	ethylene glycol, 60	5	10, 23, 45
90	ethylene glycol, 50	5	20, 50, 83
90	ethylene glycol, 40	5	20, 50, 83
80	ethylene glycol, 80	5	2, 5, 10
80	ethylene glycol, 70	5	23, 51, 81
80	ethylene glycol, 60	5	122, 281, 365
80	ethylene glycol, 50	5	1004, 1360, 2513
80	ethylene glycol, 40	5	1004, 1360, 2513
80	ethylene glycol, 30	5	1004, 1360, 2513

The activity of TtPyNP with different pyrimidine substrates was determined using reaction mixtures of 1 mM nucleoside (**1a**−**1i**), 50 mM potassium phosphate and 0.25 µg mL^{-1} *Tt*PyNP in 50 mM glycine/NaOH buffer at pH 9 and 80 °C in a total volume of 500 µL. Samples of 50 µL were withdrawn after 1, 2 and 3 min, quenched and analyzed as described above. Samples from reactions with **1a**−**1d** were quenched in 100 mM NaOH and samples from reactions with **1e**−**1i** were quenched in 200 mM NaOH.[4,5] Reference spectra for all nucleosides can be obtained from the externally hosted Supplementary Information[2] or from our previous publication about spectral unmixing.[5,6]

The inhibition of phosphorolysis by reaction products was investigated with reactions containing 1 mM **1a**, 50 mM potassium phosphate, 0−1 mM **2a** or **3** and 0.33 µg mL^{-1} *Tt*PyNP in 50 mM MOPS/NaOH buffer pH 7 at 80 °C in a total volume of 200 µL. The reactions were sampled after 1, 2 and 3 min, quenched and analyzed as described above.

The inhibition of glycosylation by 2a was investigated with reactions containing 1 mM **2a**, 2 mM **3**, 0−1 mM **1a** and 0.17 µg mL^{-1} *Tt*PyNP in 50 mM MOPS/NaOH buffer pH 7 at 80 °C in a total volume of 200 µL. The reactions were sampled at various timepoints (Table S3), quenched and analyzed as described above.

Table S3. Sampling times and volumes inhibition experiments with *Tt*PyNP in glycosylation direction.

nucleobase concentration [mM]	sampling times [min]	sampling volume [µL]	quenching solution [µL]
0.5	0.5, 1, 1.5	65	150
0.75	1, 2, 3	50	200
1	1, 2, 3	50	300
2	2, 3, 4.5	50	450

Supplementary items

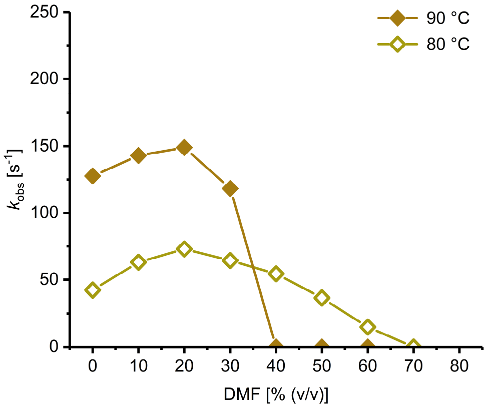

Figure S1. Activity of *Tt*PyNP in dimethyl formamide (DMF).

The activity of TtPyNP in reaction mixtures with DMF was determined by assaying for the initial rate of phosphorolysis of **1a**. Reactions with 1 mM **1a**, 20 mM potassium phosphate and 0.12–0.40 µg mL^{-1} *Tt*PyNP (0.12 µg mL^{-1} for 90 °C and 0.40 µg mL^{-1} for 80 °C) in 20 mM glycine/NaOH buffer pH 9 containing the indicated amount of cosolvent in a total volume of 500 µL were performed with samples taken after 1, 2 and 3 min. Quenching and data analysis was carried out as described above and data were fit as detailed in the metadata files available online.[2]

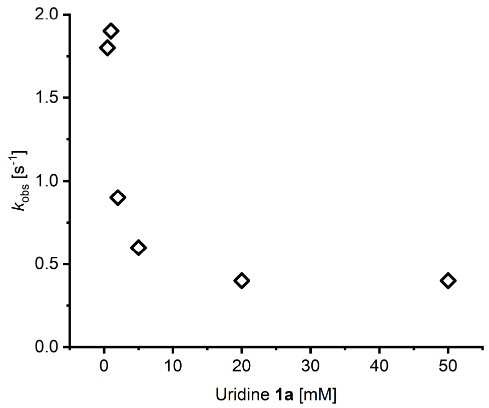

Figure S2. Kinetics of *Tt*PyNP at 40 °C and pH 9.

The kinetics of TtPyNP at 40 °C and pH 9 were analyzed using reaction mixtures of 50 mM potassium phosphate and 20 or 50 µg mL^{-1} enzyme in 50 mM glycine/NaOH buffer pH 9 at 40 °C with the indicated nucleoside concentration in a total volume of 200 or 500 µL. Reactions were run for variable times (Table S4) and samples were withdrawn, quenched and analyzed as described above (no duplicates were performed).

Table S4. Sampling times and amounts for saturation experiments with *Tt*PyNP.

nucleoside concentration [mM]	enzyme [µg mL^{-1}]	sampling times [min]	sampling amount [µL]	quenching solution [µL]
0.5	20	1, 2, 3	100	450
1	50	0.33, 0.66, 1	50	450
2	50	0.66, 1.33, 2	25	450
5	50	2, 4, 6	12	450
20	50	10, 20, 30	10	900
50	50	20, 40, 60	10	1400

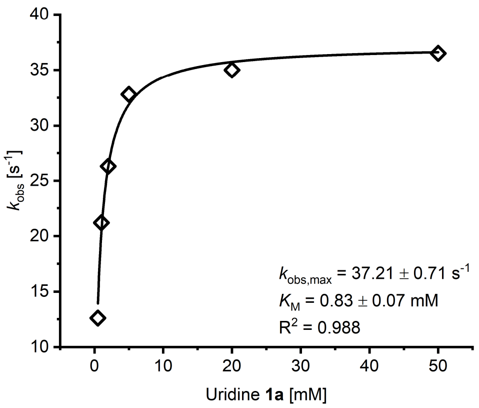

Figure S3. Saturation kinetics of *Geobacillus thermoglucosidasius* pyrimidine nucleoside phosphorylase (*Gt*PyNP).

The Michaelis-Menten-kinetics of GtPyNP were analyzed using reaction mixtures of 50 mM potassium phosphate and 1.2 or 3 µg mL^{-1} enzyme in 50 mM glycine/NaOH buffer pH 9 at 40 °C with the indicated nucleoside concentration in a total volume of 200 or 500 µL. Reactions were run for variable times (Table S5) and samples were withdrawn, quenched and analyzed as described above (no duplicates were performed). *Gt*PyNP has an extinction coefficient of 21,890 cm^{-1} M^{-1} as predicted by Protparam,[8]

thus the stock solution of 1.2 g L^{-1} had a concentration of 54.8 µM. The data were fit to the Michaelis-Menten equation (6) shown below.

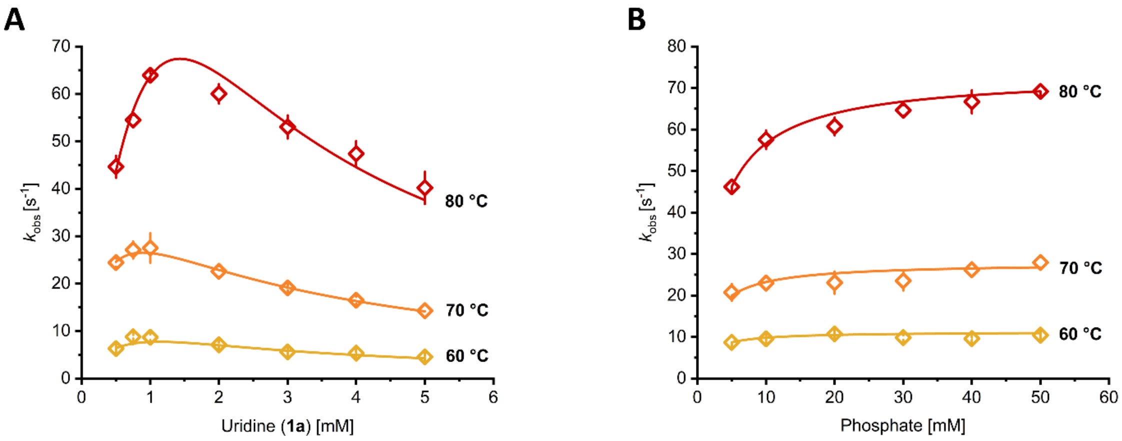

Figure S4. Apparent (but misleading) inhibition of *Tt*PyNP by nucleoside substrates. *Tt*PyNP appears inhibited by increasing concentrations of **1a** (**A**), but not by phosphate (**B**). The fluctuations in activity observed for some data in **B** are within the measurement inaccuracy.[33]

The Michaelis-Menten-type kinetics of TtPyNP were investigated through kinetic experiments with 50 mM potassium phosphate and 0.33–2 µg mL^{-1} *Tt*PyNP (0.33 µg mL^{-1} for 80 °C, 0.66 µg mL^{-1} for 70 °C and 2 µg mL^{-1} for 60 °C) in 50 mM MOPS/NaOH buffer pH 7 at the indicated temperature with the indicated nucleoside concentration in a total volume of 200 or 500 µL. The same amount of enzyme was used in all reactions for a temperature and substrate. Reactions were run for variable times and samples were withdrawn, quenched and analyzed as described above (Table S3).[2] Selected data were fit to the extended Michaelis-Menten equation accounting for substrate inhibition according to

$$k_{obs,max} = \frac{k_{obs}\,[S]}{K_M + [S] + \frac{[S]^2}{K_i}} \tag{5}$$

where $k_{obs,max}$ is the maximal observed rate constant [s^{-1}], k_{obs} is the rate constant [s^{-1}] observed at the substrate concentration $[S]$ [mM], K_M is the Michaelis-Menten constant [mM] and K_i is the inhibition constant [mM] for this substrate and enzyme combination under these conditions.

The data shown in Figure S4 yielded $K_{M,phosphate}(80°C)$ = 2.92 ± 0 mM, $k_{obs,max,phosphate}$ $(80\ °C)$ = 73.25 ± 0 s^{-1}, $K_{M,phosphate}(70°C)$ = 1.97 ± 0.68 mM, $k_{obs,max,phosphate}$ $(70\ °C)$ = 27.82 ± 1.08 s^{-1} (R^2 = 0.702), $K_{M,phosphate}(60°C)$ = 1.46 ± 0.16 mM, $k_{obs,max,phosphate}$ $(60\ °C)$ = 11.24 ± 0.28 s^{-1} (R^2 = 0.949), $K_{M,uridine}(80°C)$ = 5.92 ± 8.72 mM, $K_{i,uridine}(80°C)$ = 0.34 ± 0.50 mM $k_{obs,max,uridine}$ $(80\ °C)$ = 624.89 ± 823.49 s^{-1} (R^2 = 0.932), $K_{M,uridine}(70°C)$ = 0.38 ± 0.08 mM, $K_{i,uridine}(70°C)$ = 2.09 ± 0.31 mM, $k_{obs,max,uridine}$ $(70\ °C)$ = 49.15 ± 4.69 s^{-1} (R^2 = 0.992),

$K_{M,uridine}(60°C)$ = 0.89 ± 1.14 mM, $K_{i,uridine}(60°C)$ = 1.42 ± 1.67 mM, $k_{obs,max,uridine}$ $(60\ °C)$ = 19.95 ± 17.08 s^{-1} (R^2 = 0.676). However, it must be noted that the data in Figure S4 **do not** describe substrate inhibition, but instead a product inhibition which manifests itself in data resembling substrate inhibition.

Table S3. Sampling times and volumes for saturation experiments with *Tt*PyNP.

nucleoside concentration [mM]	sampling times [min]	sampling volume [µL]	quenching solution [µL]
0.5	0.5, 1, 1.5 or	100	300
0.75	1, 2, 3 or	75	300
1	1, 2, 3	50	300
2	2, 4, 6	50	450
3	3, 6, 9	40	450
4	4, 8, 12	30	450
5	5, 10, 15	25	450

The Michaelis-Menten-type kinetics of TtPyNP with regard to phosphate were investigated with reactions containing 1 mM **1a** and 0.25−1.33 µg mL^{-1} *Tt*PyNP (0.25 µg mL^{-1} for 80 °C, 0.66 µg mL^{-1} for 70 °C and 1.33 µg mL^{-1} for 60 °C) in 50 mM MOPS/NaOH buffer pH 7 with 5−50 mM potassium phosphate, as indicated, in a total volume of 200 or 500 µL. The same amount of enzyme was used for all reactions for a given temperature. Samples were taken after 1, 2 and 3 min. The obtained data were fit to the Michaelis-Menten equation according to

$$k_{obs,max} = \frac{k_{obs}\ [S]}{K_M + [S]} \qquad (6)$$

with definitions from above.

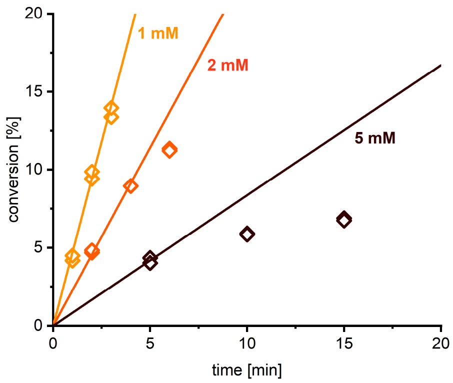

Figure S5. Non-linearity of product formation rate at higher substrate concentrations under quasi-steady-state conditions. This figure shows selected raw data of the experiments shown in Figure S4.

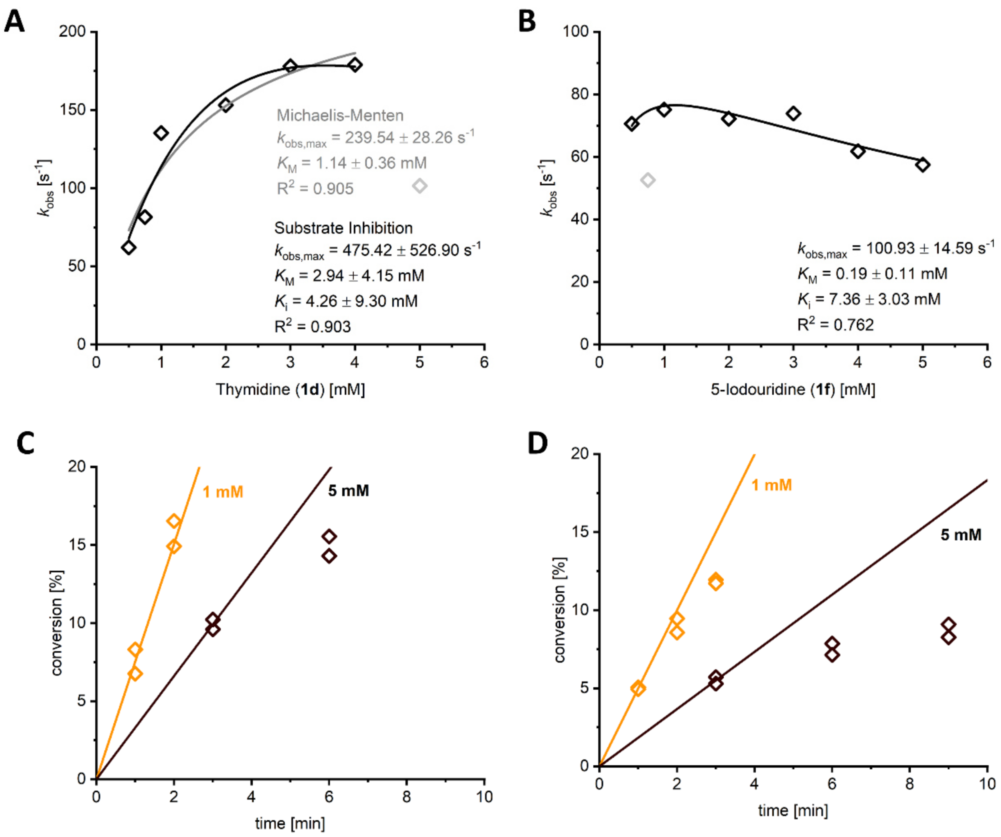

Figure S6. Saturation experiments with *Tt*PyNP and thymidine (**1d**, **A**, **C**) and 5-iodouridine (**1f**, **B**, **D**) displaying non-linear reaction courses indicative of product inhibition.

The inhibition of TtPyNP by other nucleobases was investigated with kinetic experiments employing reaction mixtures with 50 mM potassium phosphate and 0.25 µg mL^{-1} *Tt*PyNP in 50 mM MOPS/NaOH

buffer pH 7 at 80 °C with the indicated nucleoside concentration in a total volume of 200 or 500 µL. Samples were taken as described in Table S3. The data obtained[2] were fit to equation (4), but it must be noted that these data **do not** represent substrate inhibition, but instead product inhibition by the nucleobase (**2d** or **2f**).

Supplementary references

[1] A. Brand, L. Allen, M. Altman, M. Hlava, J. Scott, *Learn. Publ.* **2015**, *28*, 151–155.

[2] F. Kaspar, **2020**, DOI 10.5281/zenodo.4043929.

[3] K. Szeker, X. Zhou, T. Schwab, A. Casanueva, D. Cowan, I. A. Mikhailopulo, P. Neubauer, *J. Mol. Catal. B Enzym.* **2012**, *84*, 27–34.

[4] F. Kaspar, R. T. Giessmann, N. Krausch, P. Neubauer, A. Wagner, M. Gimpel, *Methods Protoc.* **2019**, *2*, 60.

[5] F. Kaspar, R. T. Giessmann, S. Westarp, K. F. Hellendahl, N. Krausch, I. Thiele, M. C. Walczak, P. Neubauer, A. Wagner, *ChemBioChem* **2020**, DOI 10.1002/cbic.202000204.

[6] F. Kaspar, **2020**, DOI 10.5281/zenodo.3716126.

[7] A. Cornish-Bowden, *Fundamentals of Enzyme Kinetics*, **1979**.

[8] E. Gasteiger, C. Hoogland, A. Gattiker, S. Duvaud, M. R. Wilkons, R. D. Appel, A. Bairoch, in *Proteomics Protoc. Handb.* (Ed.: J. Walker), Humana Press, **2005**, 571–607.

[9] H. Eyring, *J. Chem. Phys.* **1935**, *3*, 107–115.
